# Editorial: Next Generation Sequencing (NGS) for Rare Diseases Diagnosis

**DOI:** 10.3389/fgene.2021.808042

**Published:** 2021-12-23

**Authors:** Xiu-An Yang

**Affiliations:** ^1^ Laboratory of Genetic Engineering and Genomics, School of Basic Medical Sciences, Chengde Medical University, Chengde, China; ^2^ Hebei Key Laboratory of Nerve Injury and Repair, Chengde Medical University, Chengde, China

**Keywords:** next generation sequencing, rare diseases diagnosis, whole-exome sequencing, copy number variation, bioinformatics

## Introduction

The rapidly increasing amount of genome data provides wide opportunities to address new research questions and to translate new knowledge in clinical practice (Koriath et al., 2021). Currently, most mature areas for clinical applications of genomics include rare disease diagnostics, oncology, infectious diseases, and pharmacogenomics. We expect that the next wave is seen in improving our understanding of chronic diseases that change traditional diagnostic categories. These new disease classifications hopefully result in new, more tailored treatment and prediction options (Holzinger et al., 2015). Genomic medicine publishes high-quality research that facilitates the use of genomic information in medicine, including treatment, diagnostics, and prevention (Franks et al., 2021). With the goal to explore studies regarding clinical work that benefited from Next Generation Sequencing (NGS), this issue entitled “Next Generation Sequencing (NGS) for Rare Diseases Diagnosis” is published.

In a hemophilia A family with two male patients, Bai et al. had used NGS for preimplantation genetic testing to screen chromosome copy number variation and mutation sites. Sanger Sequencing was enrolled to verify the identified mutation sites and single nucleotide polymorphisms (SNP). Finally, a novel missense mutation, p (Phe690Leu)/c.2070C > A, occurring in exon 13 of factor VIII gene (*F8/FVIII*) was identified as a potential pathogenic mutation. Defects in F8 are documented to be the pathogenic mechanism of an X-linked recessive bleeding disorder called hemophilia A. After an F8 normal euploid blastocyst was transferred, the family had a healthy fetus, indicating the importance of NGS in assisted reproduction technology. It is worth noting that structural modeling was used for speculating the possible pathogenic mechanism of the mutation both in this study and other studies in this issue. Due to the limitation of diagnostic conditions and time, it is often difficult to verify the functions of the mutations found in possible therapeutic gene sites. Therefore, *in silico* analysis such as structural modeling plays an important role in determining the pathogenicity of mutation sites. This illustrates the importance of combining clinical practice with basic practice.

*MOCS1* (Molybdenum cofactor synthesis 1), *MOCS2* (Molybdenum cofactor synthesis 2), and *GPHN* (Gephyrin) are shown to be the pathogenic genes of molybdenum cofactor (Moco) deficiency in humans (Mayr et al., 2021). These genes, together with *MOCS3* (Molybdenum cofactor synthesis 3) are involved in Moco biosynthesis and providing cofactors to Moco-dependent enzymes (Mechler et al., 2015). However, variants in *MOCS3* have not been reported in molybdenum cofactor (Moco) deficiency in patients so far. Using trio whole-exome sequencing (Trio-WES) accompanied with Sanger sequencing validation, Tian et al. identified compound heterozygous variants in NM_014484.49 (MOCS3): c.1375C > T; p. Gln459Ter (chr20:49576754-49576754) and NM_014484.49 (MOCS3): c.325C > G; p. Leu109Val (chr20:49575704-49575704) in a proband. The identified variants were inherited from parents respectively. According to the authors, this is the first case of *MOCS3* variants causing Moco deficiency. It shows the power of NGS in the diagnosis of rare diseases, and also indicates the importance of familial segregation analysis.

Due to its rarity and sporadic characteristics, the diagnosis of rare diseases is usually quite complicated. In light of this, retrospectively investigation for a cohort of individuals who are suspected having molecular deficiency is effective for expanding both the genetic and clinic spectrum of rare diseases. Patients with disorders of nervous system (67 cases), kidney (65 cases and 69 cases respectively), and hematology (25 cases) were retrospectively studied (Ferese et al.; Kristan et al.; Stippel et al.; Wang et al.). To reveal the role of rare variants in the genotype-phenotype correlation, 67 patients with Charcot-Marie-Tooth (CMT) disease were enrolled in the study performed by (Ferese et al.). The cohort had taken copy number variation (CNV) detection and NGS panel for sequencing of 47 genes known to be associated with CMT and routinely screened in medical genetics. Both novel and previously reported causative variants were identified, broadening the phenotype expression produced by either pathogenic or undefined variants. Similarly, the other three studies are of great significance for the improvement of diagnosis of patients with unexplained rare disorders.

With the development of high-throughput sequencing technology, a large amount of sequencing data have been uploaded to relevant databases, and the mining of these public data has played an important role in the research of diseases pathogenesis including rare diseases and tumors. By means of bioinformatics, He et al. investigated the novel key genes of non-obstructive azoospermia affect spermatogenesis using RNA-seq and scRNA-seq data. Finally, 430 differentially expressed genes were identified. Of these 430 genes, C22orf23, TSACC, and TTC25, which have not been reported related to spermatogenesis are identified as hub genes. This study showed the importance of bioinformatics in disease pathogenesis.

In summary, new genes and rare variants were identified in the studies enrolled in this special topic, showing the important role of NGS in providing a tremendous opportunity to better serve our patients. The studies highlighted the association of phenotypes and the genetic heterogeneity in rare disease diagnosis. Lohmann & Klein have pointed out the importance role of NGS in the diagnosis of both rare diseases and common but heterogeneous disorders as early as in 2014 (Lohmann and Klein 2014). NGS is now widely accepted for diagnostic purposes of rare diseases. It should be noted that for most studied cases Sanger sequencing would have been as good to identify the mutations found as NGS. However, NGS can be performed in a short time at reasonable costs comparing with Sanger sequencing and can provide a large-scale potential variants (Lohmann and Klein 2014).
